# Preparation of Bacterial Cellulose/Ketjen Black-TiO_2_ Composite Separator and Its Application in Lithium-Sulfur Batteries

**DOI:** 10.3390/polym14245559

**Published:** 2022-12-19

**Authors:** Ming Yan, Chuanshan Zhao, Xia Li

**Affiliations:** State Key Laboratory of Biobased Material and Green Papermaking, Faculty of Light Industry, Qilu University of Technology (Shandong Academy of Sciences), Jinan 250353, China

**Keywords:** bacterial cellulose, separator, lithium-sulfur battery, shuttle effect, energy storage

## Abstract

Lithium-sulfur batteries (LSBs) have attracted extensive attention due to their high energy density and low cost. The separator is a key component of LSBs. An excellent LSBs separator requires not only good electrolyte wettability, but also high thermal stability, good tensile mechanical properties, green environmental protection potential and enough inhibition of shuttle effect. In this paper, composite separator Bacterial cellulose/Ketjen black-TiO_2_ (BKT) was prepared by coating the green and environmentally friendly bacterial cellulose (BC) substrate with KB-TiO_2_ material. BKT not only demonstrates higher electrolyte wettability, but also displays thermal stability and tensile resistance to enhance the safety of the battery. The high ratio of TiO_2_ and KB on the BKT surface provides chemical and physical adsorption of lithium polysulfides (LiPSs), thereby inhibiting the shuttle effect and increasing the cycle life of LSBs. The secondary current collector formed by TiO_2_ and KB can also reactivate the adsorbed LiPSs, further improving the capacity retention rate of the battery. Therefore, the LSBs assembled with the BKT separator exhibited an initial discharge capacity of 1180 mAh × g^−1^ at a high current density of 0.5 C, and maintained a specific discharge capacity of 653 mAh × g^−1^ after 100 cycles was achieved. Even at 2.0 mg × cm^−2^ sulfur areal density and 0.1 C current density, the BKT separator based battery still has an initial discharge specific capacity of 1274 mAh × g^−1^. In conclusion, BKT is a promising lithium-sulfur battery separator material. sulfur areal densities.

## 1. Introduction

The growing demand for energy storage forces the development of more advanced batteries. LSBs with higher specific capacity, energy density and lower cost are ideal alternatives to lithium ion batteries (LIB) [1,2]. However, some problems need to be solved before commercialization, i.e., the low conductivity of S and discharge products, volume expansion caused by density changer from S to Li_2_S during the redox reaction and shuttle effect [3,4,5]. Among the above shuttle effect, shuttle effect is an urgent problem. In the shuttle effect, long chain LiPSs are produced in the initial discharge process and then dissolved in the electrolyte and diffuse to the anode side through the separator to form short chain LiPSs. Moreover, the short chain LiPSs will pass through the separator and return to the cathode under the electric field [6], causing an irreversible loss of battery active materials and rapid decline of battery life [7,8]. Therefore, inhibiting the shuttle effect in LSBs and correspondingly reducing the loss of sulfur active materials is an urgent need for advancing the usage of LSBs.

The separator is one of the core components of LSBs. LiPSs generated in the reaction must pass through the separator to reach the anode [9]. Modifying the separator is a common strategy to suppress the shuttle effect [10]. As a mature commercial LIB separator, the PP separator is usually used in LSBs. Its pore size is about 200 nm and the LiPSs produced by the reaction of LSBs are about 2 nm. It is obvious that the PP separator cannot effectively inhibit the migration of LiPSs [9,11]. At present, coating the PP separator with a functional layer is the main modification of the PP separator to inhibit the shuttle of LiPSs and improve the battery life [12,13]. Generally, materials coated on the surface of PP film are carbon materials (Ketjen black (KB) [14], multi-wall carbon nanotubes (MWCNTs) [15], grapheme oxide (GO) [16], etc.) and metal oxides (Al_2_O_3_ [17,18,19], TiO_2_ [20,21], V_2_O_5_ [22,23,24], etc.). The coatings components confine LiPSs migrating to the cathode side either by physical and chemical adsorption or both, which greatly improves battery performance. Further studies are needed to promote the coating functional layer on the separator surface.

Although the shuttle effect can be effectively suppressed by modifying the PP separator, the PP separator still has some inherent disadvantages. Firstly, it has poor thermal stability, which leads to poor high temperature safety of the separator and increases the safety hazard of the battery [25,26]. Secondly, the PP separator displays low wettability which reduces the electrochemical performance of the separator to a certain extent [27,28]. Thirdly, PP of fossil origin is not green and environmentally friendly, causing harm to the environment. At present, natural polymers, especially cellulose, have been gradually used to prepare LSB separators due to their good mechanical properties, outstanding thermal stability and excellent electrolyte wettability, as well as low cost and sustainability [29]. As a kind of cellulose, BC is synthesized by different bacteria such as acetic acid bacteria and rhizobacteria through an aqueous medium. It demonstrates a nano-scale network structure, with a high degree of polymerization (4000–10,000) and a high degree of crystallinity (80–90%) characteristics [30,31], and the performance of the membrane prepared with it is better than with other cellulose. Fang [32] prepared a (PEI@BC) separator by grafting polyethyl-enemine (PEI) onto a BC membrane by a chemical grafting method. The separator had good electrolyte wettability and remained stable without observing any deformation at 200 °C. The LSBs assembled with the separator have an ultra-high initial discharge specific capacity of 1402 mAh × g^−1^ at 0.1 C current density and an excellent rate capacity of 440.5 mAh × g^−1^ at 2 C.

BC is gradually used in LSBs due to its excellent characteristics. However, few studies have used BC as a base and constructed a coating to form a new composite separator. In this paper, the KB-TiO_2_ composite with excellent conductivity and high specific surface area prepared by ultrasonic treatment is used to coat the BC membrane to form a composite separator (BKT). The separator displays good thermal stability and high electrolyte wettability. At the same time, the separator can inhibit the shuttle effect of LSBs via physical adsorption of KB with high specific surface area and chemical adsorption of TiO_2_ on LiPSs. Moreover, the secondary current collector formed by KB-TiO_2_ can reactivate the adsorbed LiPSs, thereby improving the battery performance. Taking all the above advantages, batteries using the BKT separator achieve excellent performance, making BKT a promising separator material.

## 2. Materials and Methods

### 2.1. Materials

Nano-titanium dioxide (TiO_2_, average particle size 100 nm, 99.8%) from Maclean Biochemical Co., Ltd. (Shanghai, China). BC wet film Hainan Yide Food Co., Ltd. (Hainan, China). Ketjen Black (KB, Battery level), Super p li (Battery level) and Polyvinylidene fluoride (PVDF, AR) from Taiyuan Lizhiyuan Technology Co., Ltd. (Taiyuan, China). *N*-methyl-pyrrolidone (NMP, Battery level), Dioxolane (DOL): ethylene glycol dimethyl ether (DME) = (1:1 Vol%) and Lithium-sulfur electrolyte: 1.0 M LTFSI in DOL: DME = (1:1) Vol% with 1.0% LiNO_3_ from Dongguan Kelude Innovation Technology Co., Ltd. (Dongguan, China). Sulfur (S, 99.5%) and Lithium sulfide (LiS, 99.9%) from Sinopharm Chemical Reagent (Shanghai, China).

### 2.2. Preparation of Positive Electrode Sheet

To prepare the sulfur electrode, S and KB with a weight ratio of 7:3 were thoroughly mixed in an agate mortar and ball mill in a sequent. To allow the sulfur element to infiltrate into the KB, the as prepared S/KB mixture kept in a reaction kettle was put in an electric heating blast drying oven at 155 °C for 12 h. The treated powder is further ground with mortar and is called S/C composite. First, mix S/C, super li and PVDF uniformly at a mass ratio of 7:2:1, then add an appropriate amount of NMP to grind to form slurry. The prepared slurry was coated on aluminum foil with a scraper. The modified aluminum foil was heated at 60 °C for 12 h in a vacuum drying oven to volatilize NMP and cut into a Ф 12 mm positive electrode sheet with a microtome. Three kinds of positive plates with different sulfur loading were prepared and the surface density of sulfur was 1 mg × cm^−2^, 1.5 mg × cm^−2^ and 2 mg × cm^−2^, respectively.

### 2.3. Preparation of KB-TiO_2_

A solvent mixture of absolute ethanol and deionized water with a volumetric ratio of 1:3 was prepared to disperse TiO_2_ and KB. Add 0.1 g of pre weighed TiO_2_ powder into 200 mL ethanol/deionized water solvent and conduct ultrasonic treatment for 1.5 h to form a uniform TiO_2_ suspension. Then 0.9 g of KB powder was added to TiO_2_ dispersion and the dispersion was ultrasonicated for another 2 h to obtain a homogenous a KB/TiO_2_ dispersion. Dry KB-TiO_2_ powder was finally obtained by filtration followed by freeze-drying.

### 2.4. Preparation of BKT Separator

To prepare the BKT separator, BC wet film was firstly purified: BC was immersed in 1 moL/L NaOH solution at 60 °C and washed with deionized water after one hour immersion. This combined step was repeated several times until full removal of the residual impurities in BC. Then the purified BC was vacuum-dried to obtain a BC membrane. The as-prepared KB-TiO_2_, super p li and PVDF with a weight ratio of 7:1:2 and an appropriate amount of NMP solution was mixed to form a uniform slurry. The slurry was directly coated on the BC film by using a coating machine and then placed in a vacuum drying oven at 60 °C for 12 h to obtain a KB/TiO_2_-coated BC composite membrane (BKT). The prepared BKT was cut into Ф 19 mm discs by using a microtome.

### 2.5. Li_2_S_6_ Solution Synthesis

To obtain 0.3 M Li_2_S_6_ brown-red solution, Li_2_S (0.14 g) and S powder (0.48 g) were dissolved in 10 mL of DME/DOL (volume ratio 1:1) solution under stirring at 65 °C for 48 h in an argon atmosphere glove box. The solution was further diluted to 3 mM with the same type of DOL/DME solution used for dissolution. The Li_2_S_6_ solution was further used to demonstrate the adsorption of KB-TiO_2_ to LiPSs and the inhibitory effect of BKT separator on the shuttle effect of LiPSs.

### 2.6. Characterization Techniques

The XRD of the samples was measured by an X-ray diffract meter. After placing the sample powder into the groove of the test matrix, it was flattened with a glass plate to ensure that the surface of the sample is flat. (D_8_-ADVANCE, from Bruker Co., Ltd., Bremen, Germany, voltage: 40 KV, current: 30 mA, 2θ range: 5–90°, wavelength: K<alpha>1: 1.540593, K<alpha>2: 1.54441, step size: 0.02°). Place the sample to be tested in a vacuum drying oven at 60 °C for 12 h. At the same time, place an appropriate amount of phosphorus pentoxide to prevent the generation of moisture and ensure that the sample to be tested is completely dry. After the samples were sprayed with gold, the microscopic morphology of the samples was analyzed by scanning electron microscope (SEM, Regulus8220, from Hitachi High-Tech Co., Ltd., Tokyo, Japan, EHT: 5.0KV). The element distribution on the surface of the sample was collected by an energy spectrometer (EDS, Xflash 6160, from Hitachi High-Tech Co., Ltd.). Determination of mechanical properties of samples was performed by texture analyzer (Ta. XT Plus, from Stable Micro System, Godalming, UK). Measuring the contact angle (OCA50, from Germany Dataphysics Instrument Co., Ltd., Stuttgart, Germany) between the separator and the electrolyte was carried out with a contact angle meter. The thermal stability of the sample was analyzed by thermogravimetry (TGAQ50, from TA Instrument Co., Ltd., Newcastle, DE, USA).

### 2.7. Battery Assembly and Electrochemical Testing

A CR2025 lithium battery was assembled in an argon protective atmosphere glove box with both water and oxygen content lower than 1.0 ppm. The CR2025 cell used sulfur electrode as the cathode, lithium as the anode, BKT as the separator and 1.0 M LTFSI in DOL: DME = (1:1) Vol% with 1.0% LiNO_3_ as electrolyte, respectively. At the same time, the battery assembled with KB coated BC substrate separator (BK) was used as a contrast experiment. The assembled half-cell was placed in the glove box for 24 h to ensure adequate infiltration of the electrolyte. Long-cycle and different rate (0.1–1 C) constant-current charge-discharge tests were performed on the two batteries using a Land system at room temperature, with a voltage range of 1.7–2.8 V.

In order to test the ionic conductivity of the separator, a stainless steel (SS) symmetrical battery was assembled and placed in it for 24 h, so that the electrolyte can fully soak the battery. Then, a PARSTAT electrochemical workstation (4000 A) was used to test the AC impedance of the battery at 10^5^–10^−2^ Hz and 5 mV amplitude. The following formula 1 was used to obtain the ionic conductivity: σ = L/(R_b_ × S), where σ is the ionic conductivity, L is the thickness of the separator, S is the active area of the stainless steel electrode and R_b_ is the volume resistance of the symmetrical battery [33]. The electrochemical stability of the separator was studied by line scanning voltammetry (LSV). Generally, PARSTAT is used to collect the LSV curve of SS/electrolyte immersion separator/Li half battery at a scanning rate of 5 mV s^−1^ from 1.5 to 5 V.

## 3. Results

### 3.1. Preparation and Microstructure Characterization of BKT Separator

**Figure 1:**
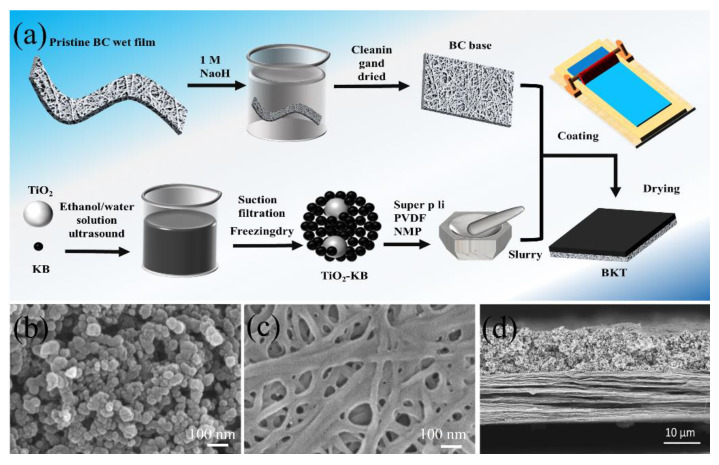
(**a**) Schematic diagram of the preparation process of BKT separator; (**b**,**c**) SEM images of the coated surface and non-coated surface of the BKT separator, respectively; (**d**) Cross section SEM of BKT separator.

Figure 1a reveals the preparation procedure for the BKT separator. A flat BC substrate was prepared by purification and further vacuum drying on the BC wet film. KB and TiO_2_ were mixed in ethanol/water solution by ultrasonic treatment, then vacuum filtration and freeze drying were performed to obtain KB-TiO_2_ composite. XRD was used to verify the crystalline structure of the KB-TiO_2_ composites with pristine KB and TiO_2_. The broad peak at 25° (Figure S1, see Supplementary Materials) revealed that KB is in amorphous state [34]. Moreover, TiO_2_ exhibits strong diffraction peaks at 2θ = 25.3°, 37.8°, 48.0°, 53.9°, 62.7° and 75.0°. These diffraction angles correspond to (101), (004), (200), (105), (204) and (215) planes contained in TiO_2,_ respectively [35]. The XRD patterns of KB-TiO_2_ composites are a combination of the pristine KB and TiO_2_, indicating the successful preparation of KB-TiO_2_ composites. The surface morphology of KB-TiO_2_ composites was observed by SEM. Figure S2 (see Supplementary Materials) shows that the material is in a loose and porous state and its abundant pores are conducive to the infiltration of electrolyte and the blocking of LiPSs. The KB-TiO_2_ was prepared into a slurry and coated on the BC substrate to obtain the BKT separator.

The morphology of the KB-TiO_2_ coated and non-coated BKT separator are shown in Figure 1b,c, separately. The uncoated BKT separator shows that the BC membrane surface is relatively flat and the long fiber network with porous structure forms a rich pore structure. A successfully KB-TiO_2_ coating was confirmed from the change of a fibril network to the porous particle distribution (Figure 1b). The secondary current collectors can reactivate the blocking LiPSs and enhance the performance of the battery [36,37]. The cross-section SEM test of the BKT separator was carried out. The test results are shown in Figure 1d. The coating composed of KB-TiO_2_ is uniformly loaded on the BC substrate and the coating is closely bound to the BC substrate. It can be seen from Figure 1d that the coatings of BKT separator are closely stacked and the BC base presents a layered structure. The thickness of the entire separator is 27 μm. The coating thickness is 12 μm.

### 3.2. Inhibition of LiPSs by BKT Separator

**Figure 2:**
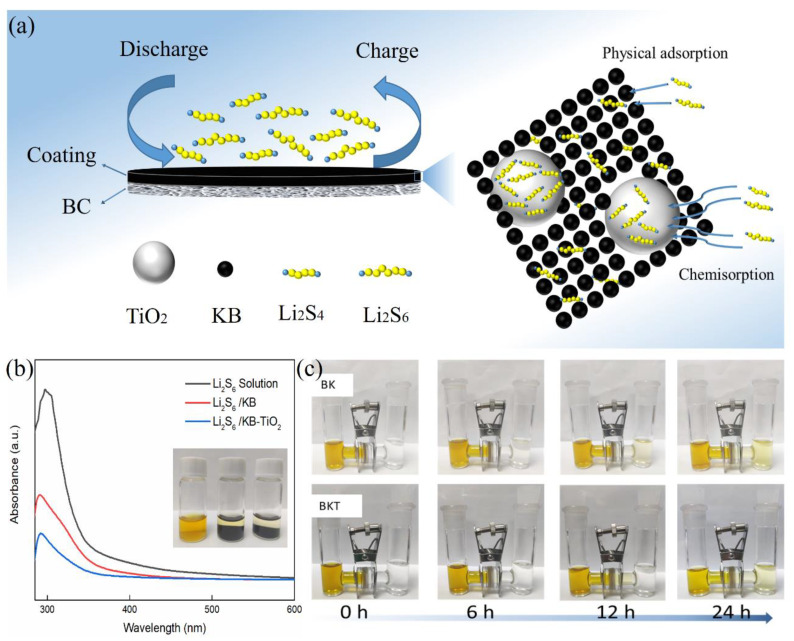
(**a**) Schematic diagram of BKT separator blocking LiPSs during charge and discharge; (**b**) KB-TiO_2_ composite material after adsorption of Li_2_S_6_ 12 h photo and its UV–Vis spectrum; (**c**) BKT separator and BK separator H-type electrolytic cell Inhibition of Li_2_S_6_ diffusion performance graph.

The working mechanism of the BKT separator is shown in Figure 2a. When LiPSs dissolved in the electrolyte pass through the secondary collector composed of KB and TiO_2_, the physical and chemical adsorption generated by KB and TiO_2,_ respectively, strongly limits the shuttle of LiPSs and improves the performance of the battery. In order to verify the adsorption capacity of KB and KB-TiO_2_ for LiPSs, a typical adsorption experiment of Li_2_S_6_ was carried out. Transfer 20 mg of KB and KB-TiO_2_ powders into vials respectively, add 2 mL of 3 mM Li_2_S_6_ DOL/DME solution, and seal the vials for 12 h. The adsorption capacity was characterized by observing the color change and UV–Vis spectrum after adsorption. The experimental results are shown in Figure 2b, the color of the blank Li_2_S_6_ sample is the darkest and the color of the solution after static adsorption of the KB sample changes obviously compared with the color of the original solution, indicating that KB has an adsorption effect on Li_2_S_6_. However, the color of the KB-TiO_2_ sample after static adsorption is lighter than that of the KB sample, indicating that KB-TiO_2_ has the best adsorption effect on Li_2_S_6_. Therefore, it is more conducive to inhibiting the shuttle effect and improving the performance of LSBs. The adsorption difference may be due to the fact that KB only provides physical adsorption; however, KB-TiO_2_ not only provide physical adsorption, but also polar molecule bonding sites for LiPSs [38]. The UV–Vis spectrum results also correlate with the adsorption experiments. A lower absorption peak of Li_2_S_6_ is observed with KB-TiO_2,_ which also indicates that KB-TiO_2_ has a higher absorption capability for LiPSs.

The concentration gradient of LiPSs on cathode and anode is the main cause of the shuttle effect of LSBs. To verify the ability of the BKT separator to suppress the shuttle effect, the diffusion behavior of Li_2_S_6_ solution was simulated in a glove box using an H-type electrolytic cell, as shown in Figure 2c. Add an appropriate amount of 3 mM Li_2_S_6_ solution and DOL/DME (volume ratio 1:1) solution to the left and right sides of the H-type electric cell. Then place the BKT and BK separators in the middle of the electrolytic cell. At 0 h, the right sides of both systems are in a clear and transparent state. At 6 h, the color on the right side of BK separator electrolytic cell changed, while the color on the right side of BKT separator electrolytic cell did not change. Over time, the color on the right side of BK membrane electrolytic cell gradually deepened, while the right side of BKT membrane electrolytic cell did not change until a slight change 24 h later, indicating that BKT membrane can effectively prevent the shuttle of LiPSs.

### 3.3. BKT Separator Performance Test

**Figure 3:**
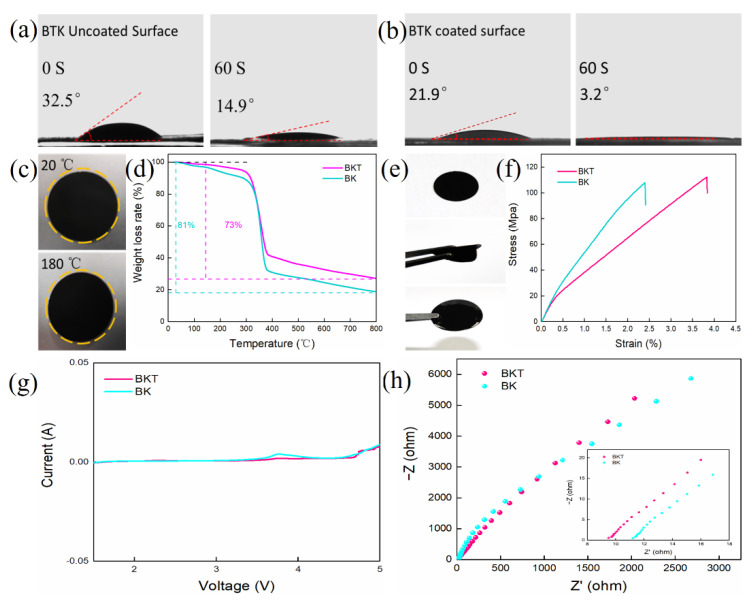
(**a**) Wettability of BKT uncoated surface to electrolyte; (**b**) Wettability of BKT coated surface to electrolyte; (**c**) Thermal dimensional stability of BKT separators at different temperatures; (**d**) TGA diagram of BKT and BK separator; (**e**) Mechanical properties of BKT separators; (**f**) BKT and BK separator Stress Strain Curve; (**g**) Linear sweep voltammetry (LSV) test for assembled (SS/separator/Li) battery.; (**h**) Nyquist diagram of assembled (SS/separator/SS) battery.

Figure 3a,b reflect the wettability of the electrolyte on the uncoated surface and the coated surface of the BKT separator using the contact angle measuring instrument. The wettability of the un-coated surface of the BKT separator to the electrolyte is reflected using a contact angle meter. The size of the contact angle reflects the wettability. The smaller the contact angle, the better the wettability of the separator. Good wettability helps to reduce the interface resistance [39]. The contact angle results against electrolyte solution are shown in Figure 3a,b. For uncoated BKT surface and coated BKT surface, the contact angle is 32.5 degrees and 21.9 degrees, respectively, at 0 s. At 60 s, the contact angle is 14.9 degrees and 3.2 degrees, respectively. It can be concluded that BKT separator has excellent electrolyte wettability. The excellent electrolyte wettability of BKT membrane is mainly attributed to the rich oxygen containing groups on its uncoated surface having affinity for electrolyte [39] and the rich pore structure of BKT coating.

Sufficiently high thermal stability of the separator is crucial to the safety of the battery. In order to explore the thermal stability of the BKT separators, a thermal shrinkage test was conducted (Figure 3c). The separator with a diameter of 19 mm is kept at 20 °C and 180 °C for 30 min respectively, then the size change of the separator is observed. It can be seen from Figure 3c that, as temperature rises, no significant dimensional change is observed for the BKT separator even at 180 °C, indicating that the BKT separator has high enough thermal dimensional stability. The excellent thermal dimensional stability of BKT is mainly due to the high crystallinity of oriented cellulose chain in BC [40]. Figure 3d shows the TGA of BKT separator and BK separator, respectively. It can be seen from the figure that, before 100 °C, two kinds of separator will experience a weight loss, which is mainly caused by the evaporation of water molecules. With the increase of temperature, the membranes degraded and the initial degradation temperature of both membranes was 299 °C. At 800 °C, the coke residue of BKT separator is 27% and that of BK separator is 19%.

The mechanical properties of the BKT separator were tested to verify the stability of the coating and the flexibility of the separator. The results are shown in Figure 3e. After the BKT separator is bent and immersed in electrolyte, the coating and BC substrate are still tightly bonded. No mechanical separation, powder or slag removal occurred, indicating that the BKT separator still has good flexibility and stability under various conditions. This excellent performance not only blocks the physical barrier of the LSBs during the cycle, but also ensures the cycle performance of the battery. The separator was subjected to tensile test using a texture analyzer. The results are shown in Figure 3f. The tensile stress at the fracture point of BKT and BK separators is 112 MPa and 107 MPa, respectively, with little difference between them. The good tensile properties can ensure battery assembly [40]. The stability of the separator during the process ensures the safety of the battery.

Figure 3g shows LSV curves of BKT separator and BK separator, respectively. LSBs will experience a chemical reaction during operation, so it is necessary to ensure that the separator has a certain chemical inertia. Therefore, linear scanning voltammetry is used to evaluate the electrochemical stability between the membrane and electrolyte. It can be seen from the figure that the response current of the two separators remains stable between 1.5–3.08 V, which can ensure electrochemical inertia in the charge discharge range of 1.7–2.8 V of lithium sulfur battery.

Next, Nyquist plots of SS/SS symmetric cells was collected to evaluate the ion conductivity of separators. Good ionic conductivity is very important to the rate performance of the battery. The test results are shown in Figure 3h, the volume resistance of the separator is represented by the real intercept, and the ionic conductivity of the separator can be obtained by equation. The volume resistances of BKT and BK separators are 9.5 Ω and 11.2 Ω, respectively. The ionic conductivity of the separator can be calculated by Formula 1. The calculated values of BKT and BK separators are 0.14 mS × cm^−1^ and 0.119 mS × cm^−1^.

In this work, KB/S composites were prepared. As shown in Figure S3 (see Supplementary Materials), the actual sulfur content in the KB/S composite obtained through TGA test is 67 wt%, close to the theoretical value (70%, mass fraction). The cathode was prepared with KB/S composite material and the battery was assembled with BKT separator to test the cycle performance and rate capability of the battery.

### 3.4. Electrochemical Characterization

**Figure 4:**
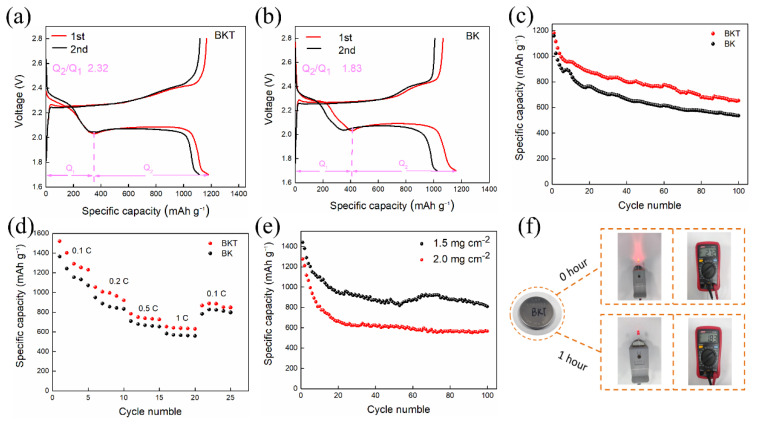
(**a**,**b**) The charge-discharge curves of BKT and BK separator-based batteries at 0.5 C current density for the 1.2 cycle; (**c**) 100-cycle long-cycle performance test of BKT and BK separator-based batteries at 0.5 C current density; (**d**) Multiplication test chart; (**e**) Long cycle diagram of BKT separator battery high sulfur loaded electrode; (**f**) Diagram of BKT separator battery lighting small bulb.

Figure 4a,b show the charge discharge curves of the first and second cycles of the battery based on BKT separator and BK separator when the current density is 0.5 C. It can be observed that the specific discharge capacities of the BKT separator based battery in the first and second cycles are 1180.8 mAh × g^−1^ and 1114.5 mAh vg^−1^, respectively, which are higher than the 1059.4 mAh × g^−1^ and 1021.8 mAh × g^−1^ of the BK separator based battery. There are two discharge plateaus in its discharge curve, among which the 2.3 V high-voltage discharge plateau corresponds to the transformation of the cyclic S_8_ molecule to Li_2_S_n_ (4 ≤ n ≤ 8). However, the low-voltage discharge plateau of the LSBs is mainly provided by the discharge capacity. First, the low-voltage discharge plateau mainly represents the reduction of Li_2_S_4_ to short-chain Li_2_S_2_/Li_2_S [41] and the charging process is mainly the transformation from Li_2_S_2_/Li_2_S to S_8_. The reason why the BKT separator battery obtains such excellent performance is mainly because the coating composed of TiO_2_ and KB can not only limit LiPSs through KB physical adsorption and TiO_2_ chemical adsorption, but also the conductive layer can be used as a secondary collector to activate the adsorbed LiPSs. Table S1 (see Supplementary Materials) shows the electrochemical performance of batteries equipped with different separators. It can be seen from the table that the BKT separator has better performance than other separators. As shown in Figure 4a,b, the phase conversion coefficient (denoted Q_2_/Q_1_) of BKT (2.32) is higher than that of BK (1.83), where Q_1_ denotes the solid-liquid conversion process of S_8_ to Li_2_S_4_ and Q_2_ corresponds to the liquid-solid-solid conversion process of Li_2_S_4_ to Li_2_S_2_/Li_2_S, implying the high sulfur utilization ability and effective LiPS conversion during the redox process [42].

Figure 4c is the 100-cycle test diagram of the BKT separator battery and the BK separator battery at a large current density of 0.5 C. It can be seen from the figure that the discharge specific capacity of both BKT separator-based and BK separator-based batteries gradually decreases with the increase in cycle numbers, but the capacity of the BKT separator battery always maintains an advantage during the cycle. The initial discharge specific capacity of the BKT separator battery is 1180.8 mAh × g^−1^, with a sulfur utilization rate of 70%. The discharge specific capacity after 100 cycles drops to 653.7 mAh × g^−1^ and the capacity retention rate is 55.4%. The initial discharge specific capacity of the BK separator-based battery is 1159.4 mAh × g^−1^, with a sulfur utilization rate of 69%. After 100 cycles, the discharge specific capacity is 535.9 mAh × g^−1^ and the capacity retention rate is 46.2%.

Figure 4d shows the magnification test diagram of BKT separator and BK separator battery. At the current rate of 0.1–1 C, the first discharge specific capacities of the BKT separator-based battery at different rates are 1520.3, 1053.9, 783.5 and 652.4 mAh × g^−1^, respectively. The BK-based batteries are 1364, 948.7, 708, 581.6 mAh × g^−1^. When the current rate is recovered from 1 C to 0.1 C, the discharge specific capacity of the BKT-based battery is 866 mAh × g^−1^ and the specific capacity of the BK-based battery is 782.6 mAh × g^−1^, the results showing that the battery assembled by the BKT separator has good reversibility. The above results show that the BKT separator inhibits the shuttle effect of LiPSs to a certain extent and shows better rate performance.

Theoretically, the LSBs has an energy density far exceeding the existing battery system. However, due to the low conductivity of the sulfur element, a large amount of inactive conductive matrix must be introduced to increase the conductivity of the LSBs. In addition, in order to achieve better electrical conductivity, the coating thickness of the positive electrode material is low and the sulfur loading is also often low, which makes it difficult for the energy density of the battery to meet people’s requirements.

Therefore, this experiment explores the electrochemical performance of BKT separator batteries at high sulfur areal densities of 1.5 mg × cm^−2^ and 2.0 mg × cm^−2^. The current density is small at 0.1 C. Compared with the charge and discharge at a large current density, the single charge and discharge time is longer and the soluble long-chain LiPSs stay in the electrolyte for a long time, resulting in a shuttle effect. The greater the possibility of this, the more the charge-discharge cycles at small current densities can verify the ability of the separator to suppress the shuttle effect. It can be seen from Figure 4e that the initial discharge specific capacity of the BKT separator battery decreases with the increase of the sulfur-loaded surface density. The initial discharge specific capacities of 0.1 C current density are 1440 mAh × g^−1^ and 1274 mAh × g^−1^, respectively, at 1.5 mg × cm^−2^ and 2.0 mg × cm^−2^ sulfur loading surface densities of electrodes. After 100 cycles, the battery still has a discharge specific capacity of 809 mAh × g^−1^ and 566 mAh × g^−1^. These results indicate that the BKT separator can also effectively inhibit the shuttle of LiPSs under the high sulfur-loading areal density of the battery, thereby enabling the LSBs to improve discharge capacity and battery energy density.

As shown in Figure 4f, the BKT separator based battery was tested for lighting the LED lamp. This figure shows that the BKT separator based battery can make the LED light bright at the beginning, but the brightness decreases significantly with time. We also monitored the open circuit potential of the battery before and 1 h after discharge. After continuously lighting the LED lamp for hours, the battery potential decreased slightly from 2.72 V to 1.83 V.

### 3.5. Characterization of BKT Separator before and after Cycling

**Figure 5:**
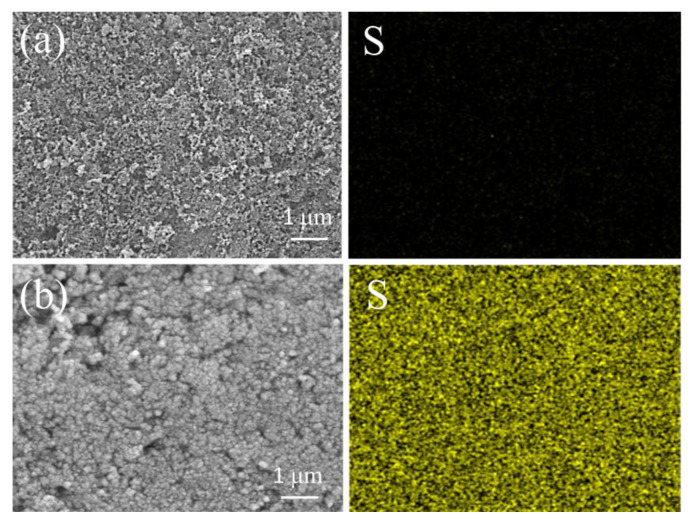
(**a**,**b**) SEM and EDS images of the BKT separator before and after 100 cycles at 0.1 C current density, respectively.

In order to further characterize the inhibitory effect of the BKT separator on the shuttle effect, SEM together with EDS tests were performed on the BKT separator before cycling and after 100 charge-discharge cycles. According to the SEM diagram before and after BKT separator cycling in Figure 5a,b, a new layer was formed on the surface of BKT separator after cycling, which indicates that the BKT separator effectively captured LiPSs during the battery charging and discharging cycle. In addition, according to the EDS diagram before and after BKT separator circulation in Figure 5a,b, sulfur was found in the separator after the charge-discharge cycle. However, no sulfur was observed on the separator prior to circulation. The appearance of sulfur element further demonstrated the retention of LiPSs on the BKT membrane.

## 4. Conclusions

In conclusion, in this paper, green BC is used as a substrate, and KB-TiO_2_ material is coated on its surface to form a BKT separator, which is used to suppress the shuttle effect of LSBs. The BKT separator not only has excellent thermal stability and good wettability to electrolyte, but also shows excellent electrochemical performance of LSBs. The results show that the initial discharge specific capacity of the battery based on the BKT separator with 0.5 C high current density is 1180 mAh × g^−1^ under the surface density of 1 mg × cm^−2^ sulfur carrying electrode. At the same time, the initial discharge specific capacity of the battery based on BKT separator at 0.1 C low current density is 1440 and 1274 mAh × g^−1^, respectively, under the high sulfur carrying surface density of 1.5 and 2 mg × cm^−2^ electrodes. The above results prove that the separator can suppress the shuttle effect of LSBs well and demonstrates a good application prospect for the separator.

## Data Availability

The data presented in this study are available on request from the corresponding author.

## Supplementary Materials

The following supporting information can be downloaded at: https://www.mdpi.com/article/10.3390/polym14245559/s1, Figure S1: XRD pattern of KB-TiO_2_ composite.; Figure S2: SEM image of KB-TiO_2_ composite.; Figure S3: TGA diagram of KB/S composites; Table S1: Performance comparison between batteries with BKT separator and batteries with other separators. References [43,44] are cited in the Supplementary Materials.
