# Supplementary material for: Preparation of Bacterial Cellulose/Ketjen Black-TiO2 Composite Separator and Its Application in Lithium-Sulfur Batteries
Source: Polymers (Basel). 2022 Dec 19;14(24):5559. doi: 10.3390/polym14245559 (PMC9788007; doi:10.3390/polym14245559)
Supplement: Supplementary file 1 [file polymers-14-05559-s001.zip › polymers-2095738-supplementary.pdf]

polymers

MDPI

Supplementary Materials: Preparation of Bacterial Cellulose/Ketjen Black-TiO_2 Composite Separator and Its Application in Lithium-Sulfur Batteries

Ming Yan, Chuanshan Zhao * and Xia Li

State Key Laboratory of Biobased Material and Green Papermaking, Faculty of Light Industry, Qilu University of Technology (Shandong Academy of Sciences), Jinan 250353, China
* Correspondence: ppzcs78@163.com

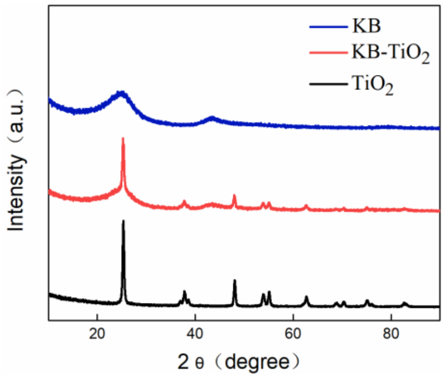

Figure S1. XRD pattern of KB-TiO_2 composite.

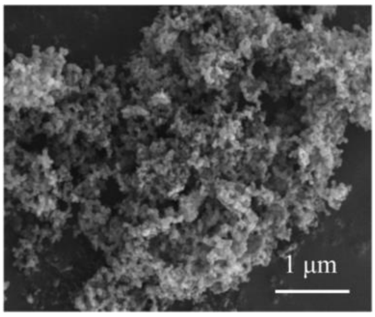

Figure S2. SEM image of KB-TiO_2 composite.

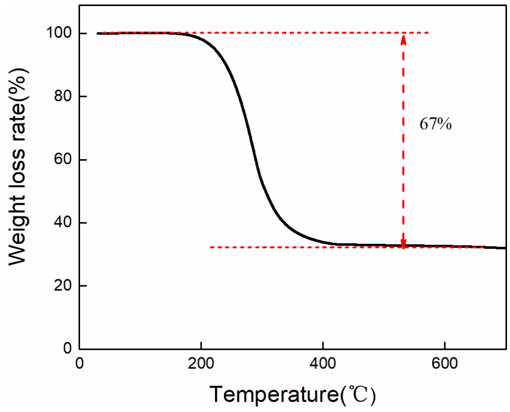

Figure S3. TGA diagram of KB/S composites.

Table S1. Performance comparison between batteries with BKT separator and batteries with other separators.

Sample	Sulfur surface density (mg/cm^2)	Rates	Initial capacity（$mAh \times g^{-1}$）	References
H-TiO_2@rGO@PP	1.6	0.3C	1037.9	Ref. [43]
KB@ZIF-8/PP	1.2	0.1 C	1235.6	Ref. [14]
CBC/TiO_2 modified separator	1.5	0.2C	1314	Ref. [44]
BKT	1.0	0.5C	1180	This paper
